# Acute Morphine Treatments Alleviate Tremor in 1-Methyl-4-Phenyl-1,2,3,6-Tetrahydropyridine-Treated Monkeys

**DOI:** 10.1371/journal.pone.0088404

**Published:** 2014-02-10

**Authors:** Ting Yan, Joshua Dominic Rizak, ShangChuan Yang, Hao Li, BaiHui Huang, YuanYe Ma, XinTian Hu

**Affiliations:** 1 Key Laboratory of Animal Models and Human Disease Mechanisms, Kunming Institute of Zoology, Chinese Academy of Science, Kunming, Yunnan, China; 2 Kunming Primate Research Center, Kunming Institute of Zoology, Chinese Academy of Science, Kunming, Yunnan, China; 3 Yunnan Key Laboratory of Primate Biomedical Research, Kunming, Yunnan, China; 4 University of Chinese Academy of Science, Beijing, China; Emory University, United States of America

## Abstract

Parkinson’s disease (PD) is a chronic and progressive neurodegenerative disorder associated with decreased striatal dopamine levels. Morphine has been found to elevate dopamine levels, which indicates a potential therapeutic effect in PD treatment that has not been investigated previously. To evaluate this hypothesis, an investigation of the acute effects of morphine on PD symptoms was carried out in male rhesus PD monkeys that had been induced with MPTP. All MPTP induced monkeys displayed progressive and irreversible PD motor symptoms. The behavioral response of these animals to morphine and L-Dopa were quantified with the Kurlan scale. It was found that L-Dopa alleviated bradykinesia, but did not significantly improve tremor. In contrast, acute morphine alleviated tremor significantly. These results suggested that, compared to L-Dopa, morphine has different therapeutic effects in PD therapy and may act through different biological mechanisms to alleviate PD symptoms.

## Introduction

Parkinson’s disease is a progressive neurodegenerative disorder characterized clinically by tremor, bradykinesia and rigidity [1]. This pathology is associated with the loss of dopaminergic neurons in the substantia nigra pars compacta (SNc) and the resultant dopamine (DA) depletion in the striatal nerve terminals [2], [3]. In PD patients, a large proportion (50%) of dopaminergic neurons in the SNc and approximately 80% of striatal dopamine levels have been lost [4], [5], [6]. Due to the large loss of brain dopamine, therapies of PD are mainly focused on the elevation of brain dopamine levels. Thus, the mainstay therapy is dopamine replacement [7]. l-3,4-dihydroxyphenylalanine (L-Dopa), the DA precursor, is a classic drug used in PD patients. The enzyme dopa decarboxylase converts L-Dopa to DA, which exogenously increases the levels of brain dopamine. Other drugs, such as monoamine oxidase B inhibitors, dopamine agonists and N-methyl-D-aspartate antagonists, are also used alone or in combination with L-Dopa in the treatment [8].

Morphine is an opiate alkaloid which is clinically used for pain management [9]. In addition to analgesia, morphine has an ability to elevate dopamine levels in the mesolimbic dopamine system [10], [11]. Morphine treatments elevate dopamine levels by mimicking the effects of endogenous morphine at µ, κ and /or δ opioid receptors [12]. Among these receptors, stimulation of the µ opioid receptor in the ventral tegmental area (VTA) has displayed the strongest effects in activating the mesolimbic dopamine system [11], [13], [14], [15], [16]. This is dependent on the high affinity of the µ opioid receptor for morphine [11]. Stimulation of the µ opioid receptor hyperpolarizes γ-amino-butyric acid (GABA) interneurons in the VTA, thereby inhibiting GABA release [17]. This leads to activation of dopaminergic neurons and enhancement of dopamine release by disinhibition [18], [19], [20]. These interactions between opioid receptors and dopamine in the mammalian brain indicates there is a potential therapeutic effect of morphine in PD treatment.

Previous studies investigating the interactions between dopamine and opioid receptors [21], [22], [23], [24], [25] have reported that morphine can decrease levodopa-induced dyskinesia [22], [23], [26] and induce akinesia [23], [26], [27]. However, no therapeutic effect of morphine on PD symptoms has been reported. Nonetheless, the ability of morphine to elevate brain dopamine levels through different mechanisms than L-Dopa and the different pathological mechanisms that underlie different clinical PD symptoms [28] suggest that morphine may have different effects on the PD symptoms than L-Dopa. These differences may have important implications in PD treatments as well as in understanding their mechanisms of action. To elucidate the effects of morphine on PD symptoms, a number of clinical symptoms were investigated separately in this study using a rhesus macaque PD model.

1-methyl-4-phenyl-1,2,3,6-tetrahydropyridine (MPTP) is a neurotoxin that selectively targets dopaminergic cells and has been found to produce parkinsonian syndromes in rodents, primates and human [6], [29], [30]. MPTP and its metabolite 1-methyl-4-phenylpyridinium (MPP^+^) are generally thought to inhibit mitochondrial complex I of the electron transport chain and generate reactive oxygen species, which leads to an apoptotic response in dopaminergic neurons [31], [32]. In primates, MPTP treatments can replicate almost all of the motor symptoms of human PD, such as rigidity, bradykinesia, as well as tremor, which has been the most difficult symptom to reproduce in many PD animal models [33], [34]. Furthermore, MPTP treatments have also been shown to reproduce other classic changes that occur in PD patients, such as cognitive, biochemical, and histological changes [35], [36]. In addition, symptoms induced by MPTP are ameliorated with pharmacological treatments of L-Dopa and other DA agonist drugs [37]. All these make the chronic MPTP primate model an ideal PD model to examine whether morphine can attenuate PD symptoms. L-Dopa, because it is the most effective symptomatic therapy [8], was used in this study as a positive control and for the comparison of morphine treatment effects.

## Materials and Methods

### Ethics statement

The five monkeys *(macaca mulatta*) (6–8 years old, 7–9 kg) from the breeding colonies at the Kunming Institute of Zoology (KIZ) were used in this study. These animals were housed in a room (6 * 6 * 5 m) under controlled conditions of humidity (60%), temperature (20°C ±2°C) and light (12-hour light/12-hour dark cycles: lights on at 7:00 A.M.). Each monkeys had its own cage (80 * 80 * 80 cm ) and could communicate with others. Two toys (such as hollow balls, for child use) were put in each cage and were changed every week. In addition, videos were played three times per week to enrich animals’ environment. Food (commercial monkey biscuits) and water were available *ad libitum*. In addition, the animals were fed with fruits and vegetables once daily.

Care and treatment of the monkeys were in strict accordance with the guidelines for the National Care and Use of Animals approved by the National Animal Research Authority (P.R.China) and the Institutional Animal Care and Use Committee (IACUC) of Kunming Institute of Zoology (approval ID SWYX-2010010). All efforts were made to minimize suffering. For example, in addition to the daily fruits and vegetables feeding, we fed monkeys with extra fruits before and after each injection. Therefore, these animals were trained to receive the injections readily (no anesthetics were used). All animals behavior was recorded using a digital video recorder, which helped the monitoring. The routine veterinary care was done by professional keepers and veterinarians.

After this study, no animals were sacrificed. These monkeys were carefully housed and were subjected to other studies.

### MPTP treatment

All five monkeys received progressive MPTP (Sigma, St Louis, MO, USA) treatment (intramuscular injection, 0.2 to 0.4 mg/kg, injections spaced by 2 to 3 days) to develop the chronic PD model. Injections were repeated until motor symptoms were fully expressed (see 'Behavioral analysis' section below).

After the MPTP administration, the five MPTP-treated monkeys were divided into two groups for L-Dopa treatment (n = 2, group I) and morphine treatment (n = 3, group II).

### L-Dopa treatment

Each monkey in group I received L-Dopa treatments for the period of one month. The L-Dopa dosage (*d*) was determined according to the Meeh-Rubner conversion formula: A = kW^2/3^; where A is the total body surface area, k is the Meeh-Rubner constant (k_monkey_ = 11.8, k_human_ = 10.6) and W is the animal’s weight. The dose ratio for the monkeys was *d*_monkey_ (mg)  = *d*_human_ (mg) × (*A*_monkey_/*A*_human_). L-Dopa was given at 10 mg/kg for the first week and was then increased by 5 mg/kg per week. The animals were given treatments twice a day in the form of pulverized tablets (Kunming Quanxin Biological Pharmacy Company, Yunnan, China) dissolved in orange juice.

During the evaluation of the long-term effects of L-Dopa on PD symptoms in group I monkeys, the monkeys in group II were used as controls to exclude the effects of any natural recovery in the animals following the completion of the MPTP administration. Group II animals were not given any drugs for one month period to measure the extent of the natural recovery.

### Morphine treatment

After the one-month control period, group II was subjected to morphine treatments. Each monkey in group II (n = 3) received a single intramuscular morphine injection (morphine hydrochloride (C_17_H_19_NO_3_·HCl·3H_2_O); Sheng Yang 1st Medical Company, Sheng Yang, China; 5 mg/kg) [38] to examine whether morphine can attenuate motor symptoms. A single dosing was used to avoid repeated exposures to morphine, which may lead to dependence and addiction [16].

### Behavioral analysis

All monkeys’ behavior was recorded in their home cages using a digital video recorder. In order to obtain monkeys’ PD scores and determine the severity of parkinsonism, the behavior was quantified by using the rating scale proposed by Kurlan [39]. The scale includes 7 items rated between 0 and 2 or 4, with a total score of 20. It takes into account classical motor symptoms (tremor, bradykinesia, posture and arm posture)as well as spontaneous activities (arm movements) and other activities (balance and defense reaction). A score ≥ 15 corresponds to the full expression of parkinsonism, similar to stage IV of the scale of Hoen and Yahr [40].

During the induction of the PD model using MPTP, each monkey was recorded once per day (one hour) to obtain its daily total PD score (the sum of all the scores of the 7 symptoms mentioned above: tremor, bradykinesia, balance etc.). This allowed for the evolution of PD symptoms to be followed. During both the L-Dopa and morphine treatments, monkeys’ behavior were recorded twice per day: (1) to obtain the daily total PD scores in order to assess the chronic effects of L-Dopa treatments on PD. This was the first one-hour recording which was carried out before the monkey received the treatment for the day; (2) to obtain the score of each symptom in order to evaluate the acute effects of L-Dopa and morphine on each individual PD symptom. This was the second one-hour recording which was carried out right after the administration of the drug.

### Data analysis

Each behavioral recording was split equally into four parts (15 min each), and each part served as an unit for acquiring PD scores.

In order to follow the evolution of PD symptoms and assess the chronic effects of L-Dopa treatments on PD, the daily total PD scores were obtained from the recordings during the MPTP induction period and the first one-hour observations (see 'Behavioral analysis' section above) during the L-Dopa treatments. All four parts of each of those recordings were scored and then averaged to obtain the daily total score. During the one-month L-Dopa-treatment period, the difference in the daily total scores between group I (monkeys were given L-Dopa) and group II (monkeys were not given any drugs) was assessed by a independent *t*-test and analysis of variance analysis.

To evaluate the acute effects of both the L-Dopa and morphine treatments on each individual PD symptom, the second post-administration one-hour recording for L-Dopa and morphine treatments were evaluated. The first 15-min section of each recording was excluded to account for immediate drug metabolism and the three remaining 15-min sections were evaluated. The change of each PD symptom was calculated individually using the same comparisons between the scores before and after the drug administration. The acute effect of morphine and L-Dopa was scored using measurements on the same day, respectively. Whereas, each PD symptom was assessed for the chronic effect of L-Dopa by comparing the scores after the last L-Dopa treatment (day 31) with the score prior to any L-Dopa administration (day 0). Paired *t*-test and analysis of variance analysis was used.

Three levels of significance were considered: *P*<0.05, *P*<0.01 and *P*<0.001. All data were presented as the mean ± standard error of mean.

## Results

### Evolution of symptoms

**Figure 1:**
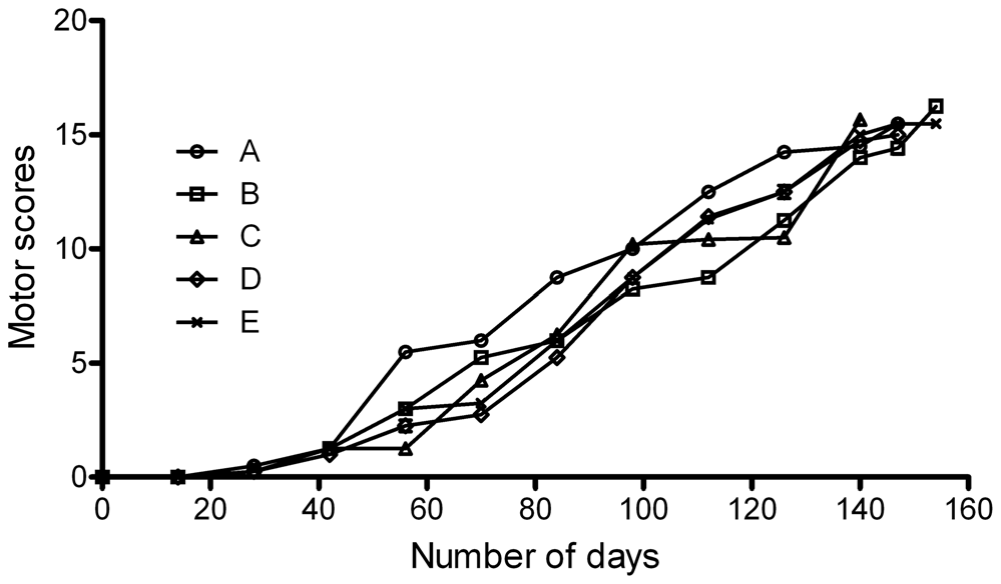
Evolution of daily motor scores for each monkey (group I and II) under MPTP induction. The animals received progressive MPTP injection (0.2 to 0.4 mg/kg, i.m.) every 2 to 3 days until motor symptoms were fully expressed ( a total Kurlan score >15).

Monkeys (groups I and II) received 20 to 22 (mean 21.2 ± 0.9) weeks of MPTP injections for a cumulative dose of 11 to 12.6 mg/kg (mean 11.9 ± 0.8 mg/kg). The pattern of parkinsonism symptom development was similar in all MPTP-treated monkeys (5/5). In the first four weeks, monkeys gradually displayed more daily naps and less movement. Subsequently, monkeys displayed increasing bradykinesia with a flexed posture. Other behaviors, such as rigidity, freezing, loss of balance, impaired defensive reactions and tremor were also observed in the animals. All symptoms gradually worsened over time until these animals all developed motor symptom scores larger than 15 (full expression) (Fig. 1).

**Figure 2:**
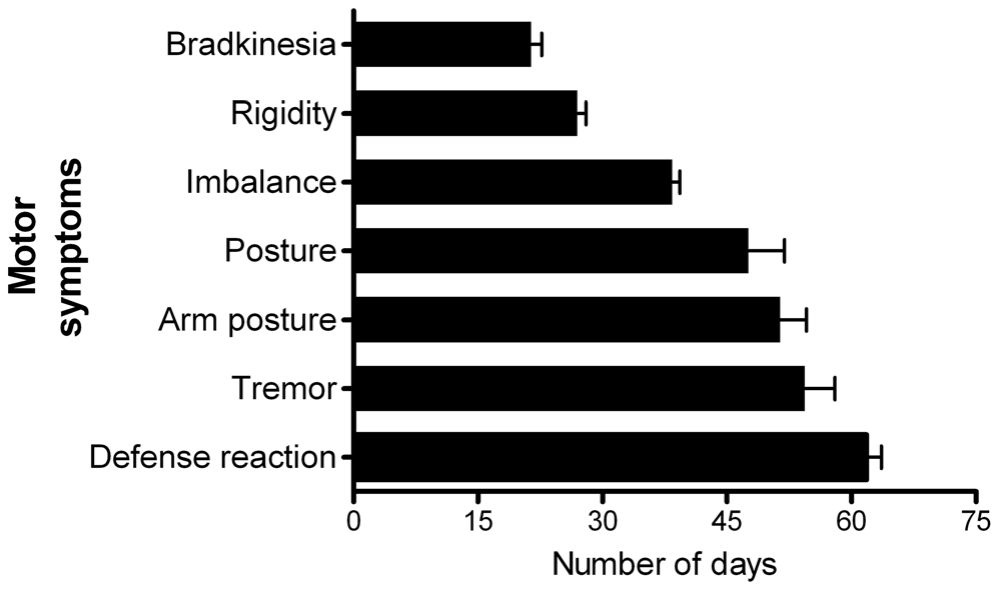
The appearance of individual PD symptoms in monkeys given MPTP intoxication. This graph presents the average day of appearance for each symptom. Error bars indicate the SEM for appearance of each symptom (n = 5).

The first motor symptom to appear was usually bradykinesia, while tremor and impaired defense reaction came out lastly (Fig. 2). Other early appearing symptoms included rigidity and loss of balance (Fig. 2). This pattern of symptom development was consistent with a previous study that developed PD symptoms using MPTP induction [41]. This consistency between the macaque PD model developed here, which displayed all important PD symptoms, and those developed earlier suggested that it was suitable to evaluate further drug administration (L-Dopa and morphine, respectively).

### Chronic effects of L-Dopa treatments measured by total PD scores

**Figure 3:**
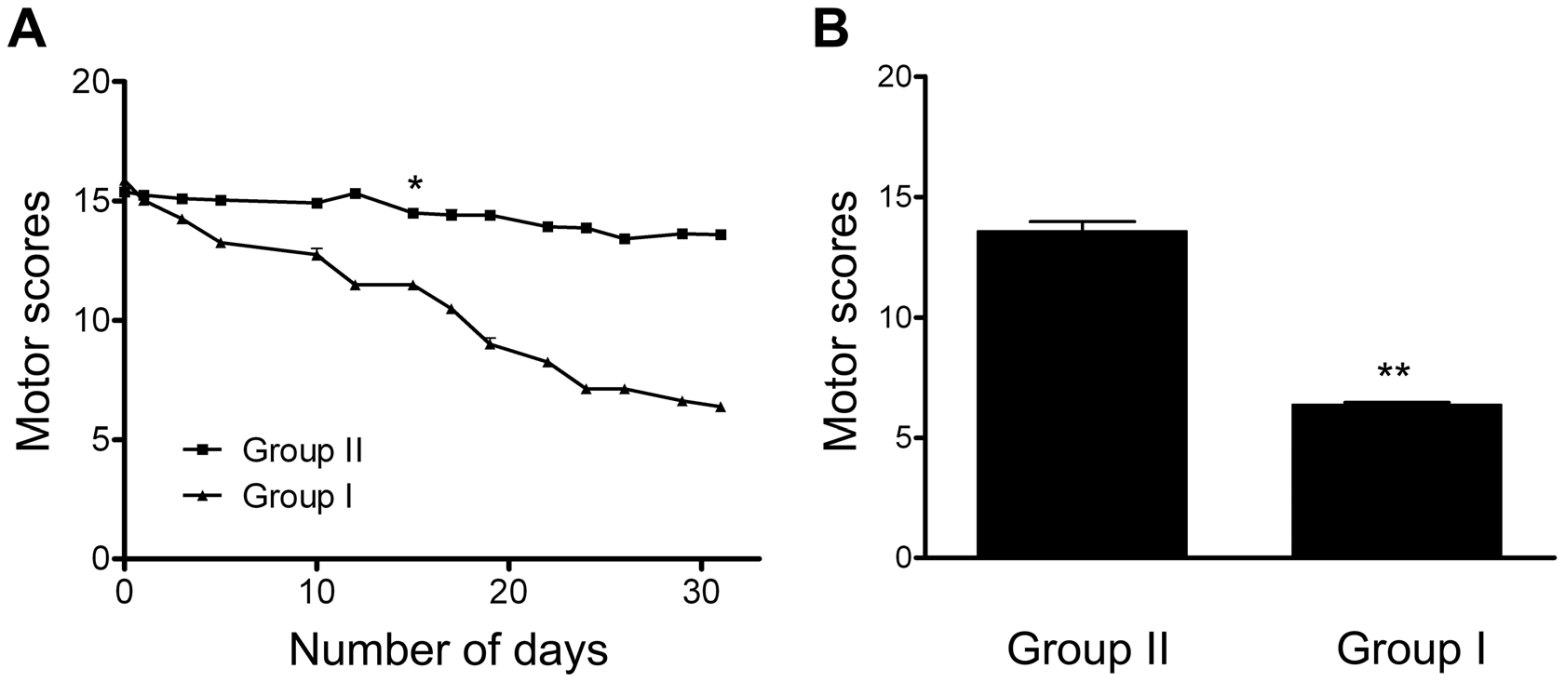
Chronic effects of L-Dopa treatments on total motor scores. (A) Evolution of daily motor scores during L-Dopa treatment. Timelines are aligned such that day 0 corresponds to the day on which the MPTP intoxication was stopped for each animal. Monkeys in group I received L-Dopa from day 1 and scores were taken from recordings prior to the daily L-Dopa administration. Significant differences between the two groups appeared at day 15 and continued into the later days (day 16–30). (B) Total Kurlan scores on day 31 of the two groups. Monkeys in group I received L-Dopa and displayed improvement in their total PD scores compared with the control group. Group I, n = 2; group II, n = 3. ^*^P< 0.05, ^**^P<0.001. Data are presented as mean ± SEM.

After one-month of L-Dopa treatments, the PD symptoms induced by MPTP in group I were found to be significantly alleviated. This was reflected by a significant decrease in the daily total PD scores on the parkinsonian rating scale that began on day 15 of treatment (Fig. 3A). The total scores improved by 53% (P < 0.001) after the one month administration of L-Dopa (day 31) (Fig. 3B). The improvements observed in group I (n = 2) were due to L-Dopa treatments; no statistically significant decrease in PD scores were observed in group II monkeys, which did not received any treatment and served as a control for the one-month period of L-Dopa administration, suggesting that little natural recovery from the MPTP period occurred over the one-month period. The result that PD symptoms in group I responded to L-Dopa therapy was consistent with previous studies [42], [43] and further confirmed the success of the development of the PD model in this study.

### Comparison of the effects between morphine and L-Dopa treatments on each symptom

**Figure 4:**
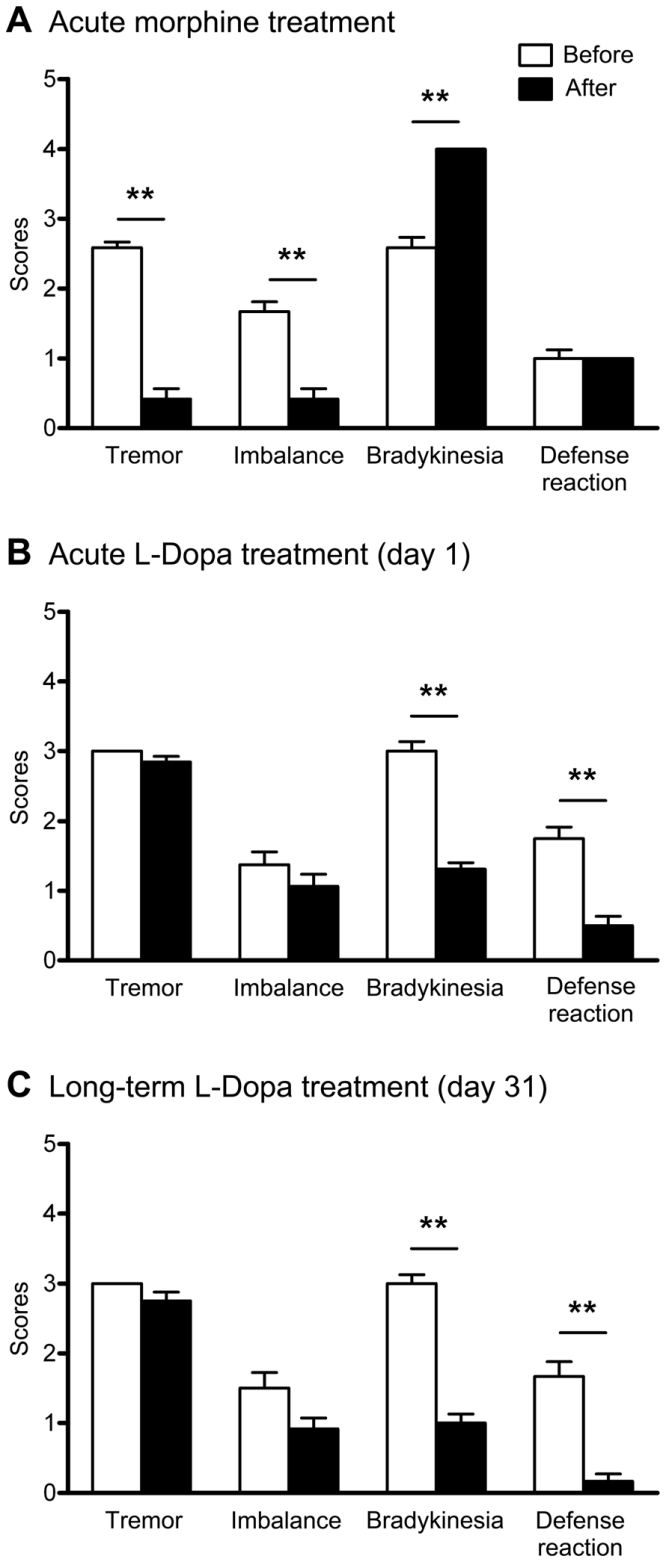
Behavioral scores of tremor, imbalance, bradykinesia and defensive reaction in the two groups before and after the administration of morphine or L-Dopa. Behavioral measurements were taken before and immediately after the administration of drug. (A) Behavioral scores after an acute dose of morphine. Tremor and loss of balance were significantly attenuated, while bradykinesia became much more prominent in group II monkeys. (B) Behavioral scores after the first dose of L-Dopa. Monkeys in group I displayed short-term amelioration of bradykinesia and defensive reaction on the first day of L-Dopa therapy. Tremor did not statistically change. (C) Behavioral scores after the last dose of L-Dopa. Monkeys in group I received L-Dopa treatments for 31 days and displayed amelioration of bradykinesia and defensive reaction on the last day (day 31) of L-Dopa therapy. No significant differences were found for tremor. Group I, n = 2; group II, n = 3. ^**^P<0.001. Data are presented as mean ± SEM.

After a single morphine injection, the three group II monkeys displayed some therapeutic responses to morphine which were noted in regards to tremor and imbalance (Fig. 4A). Before the morphine injection, the monkeys displayed a severe level of tremor and had major lapses in balance during the majority of the time being analyzed. After the application of morphine, the tremor and loss of balance significantly improved (P < 0.001). However, bradykinesia worsened (akinesia) after the injection of morphine compared to before the injection (P < 0.001). Other motor symptoms, such as defensive reaction, did not improve (Fig. 4A).

In contrast, the effects of L-Dopa treatments on each motor symptom were found to be different from those of the morphine treatments. As group II monkeys only received one morphine treatment to avoid potential addiction complication, the behavioral scores were compared to the first day scores for each symptom in group I monkeys treated with L-Dopa. On the first day that group I monkeys received L-Dopa treatment, animals displayed temporary improvements in bradykinesia and their defensive reactions, which lasted for about one hour (P<0.001) (Fig.4B). However, no change in tremor was found (Fig.4B). Similar to the L-Dopa effects on day 1, bradykinesia and defensive reaction behaviors were found to be alleviated on the last day of the L-Dopa treatments, both prior to and after administration (day 31), however there was no significant improvement on tremor observed (Fig. 4C). This suggested that the effects of the acute morphine treatment on PD symptoms were different from the effects of L-Dopa in either acute and chronic treatments.

## Discussion

Morphine can be endogenously synthesized and can increase DA firing rate through disinhibition [44], [45]. In addition, upregulation of neuronal endogenous morphine-like compound immunoreactivity was found in human PD [46]. These facts suggest morphine may have potential therapeutic relevance in PD treatment as dopaminergic neuron loss is related to Parkinson’s disease etiology. However, the use of morphine as a potential therapeutic for PD has not been reported. In this study, an initial investigation into the acute effects of exogenous morphine on PD symptoms was carried out. A rhesus macaque chronic PD model was established through MPTP intoxication. The evolution of PD symptoms were observed to be consistent with other progressive PD models [41], [47], [48]. Following which the animals were treated with either L-Dopa or morphine, four PD symptoms, tremor, bradykinesia, imbalance and defensive reaction, were found to be differentially affected by either morphine or L-Dopa. However, we would concentrate on the changes of tremor and bradykinesia as the two symptoms were the primary PD symptoms. Most notably, morphine treated PD monkeys displayed significant improvements in tremor. This beneficial effect of morphine has not been reported before and indicates a potential therapeutic effect of morphine in PD treatment.

Interestingly, the therapeutic effect of morphine was different than that of L-Dopa, the classical PD symptomatic therapy. On the first day of L-Dopa treatment, L-Dopa-treated monkeys displayed temporary improvements on bradykinesia, which was not witnessed in the morphine-treated monkeys. However, L-Dopa did not display the same improvement that morphine treatment had on tremor. After one-month of L-Dopa treatment, these differences persisted between the chronic effects of L-Dopa and the acute effect of morphine. It is noteworthy that the L-Dopa chronically treated monkeys displayed lasting improvements on bradykinesia in comparison to acute morphine or acute L-Dopa treatments. Nonetheless, the chronic L-Dopa treatment was not found to improve tremor, which was consistent with previous studies [42], [43]. The different effects of acute morphine on PD symptoms in comparison to the acute and long-term effects of L-Dopa suggest that morphine may have an alternative or complementary role in the treatment of PD symptomology, especially as it pertains to tremor.

The different therapeutic effects of morphine and L-Dopa may due to different mechanisms in elevating brain dopamine levels. L-Dopa is DA precursor and converts to dopamine directly by the enzyme dopa decarboxylase. In addition to this direct way of increasing brain dopamine, L-Dopa has also been demonstrated to increase production of dopamine in nigral dopaminergic neurons [49], [50] by exciting nigral dopaminergic neurons through AMPA/kainate receptors and other receptors [42], [43], [44]. However, in contrast, morphine elevates brain dopamine levels by stimulating µ opioid receptor, which inhibits GABA release and consequently enhances dopamine release [17], [19].

In addition to the different mechanisms of elevating brain dopamine between morphine and L-Dopa, the differences in the neuronal bases between tremor and bradykinesia could be another possible reason to explain their different therapeutic effects. In PD patients and animals, dopaminergic cell loss in SNc and dopamine depletion in the striatum, particularly in the dorsolateral putamen [51], are strongly linked to bradykinesia [52]. While tremor may result from a pathological interaction between the basal ganglia loop and the cerebello-thalamo-cortical circuit [53], [54]. The cerebello-thalamo-cortical circuit receives signals from dopamine-depleted basal ganglia (pallidal) through the primary motor cortex [28], [54], [55], [56] and then produces the tremor and controls its amplitude [57]. Considering the different effects between morphine and L-Dopa, the brain structures mentioned above might have different responses to the two drugs.

Altogether, a novel effect of morphine on PD symptoms has been observed in this study by using a rhesus macaque chronic PD model, which was induced by MPTP intoxication and was confirmed in two animals who responded to L-Dopa therapy. The therapeutic effect of morphine is different from the effects of L-Dopa, most notably by reducing tremor where L-Dopa does not. This novel effect of morphine provides some information on opioid receptor related PD therapy [58], [59], which suggests a potential new strategy for PD treatment in the future.
